# Predictors of cannabis use among first-time justice-involved youth: A cohort study

**DOI:** 10.1016/j.drugalcdep.2021.108754

**Published:** 2021-05-21

**Authors:** Marina Tolou-Shams, Johanna B. Folk, Brandon D.L. Marshall, Emily F. Dauria, Kathleen Kemp, Yu Li, Daphne Koinis-Mitchell, Larry K. Brown

**Affiliations:** a University of California, San Francisco, Department of Psychiatry and Behavioral Sciences, 1001 Potrero Ave, San Francisco, CA, 94110, USA; b Brown University School of Public Health, Department of Epidemiology, 121 South Main Street, Providence, RI, 02903, USA; c The Warren Alpert Medical School of Brown University, Department of Psychiatry and Human Behavior, 222 Richmond St, Providence, RI, 02903, USA; d The Warren Alpert Medical School of Brown University, Department of Pediatrics, 222 Richmond St, Providence, RI, 02903, USA; e Rhode Island Hospital, Division of Child and Adolescent Psychiatry, Coro West, 1 Hoppin Street, Providence, RI 02903

**Keywords:** Cannabis use, Cannabis expectancies, Justice-Involved youth

## Abstract

**Background:**

Justice-involved youth use cannabis at higher rates than their same-aged peers increasing likelihood of adverse behavioral health consequences and continued legal involvement. This study examined individual level predictors of early onset use cannabis use (<13 years of age) and cannabis use initiation in the 12 months following first court contact.

**Methods:**

Participants were 391 first-time justice-involved youth (56.9 % male; *M*_age_ = 14.6 years; 32.1 % White, 11.1 % Black, 14.7 % Other/Multi-racial, 42.2 % Latinx) and an involved caregiver (87.2 % female; *M*_age_ = 41.0 years). Baseline assessments captured individual level factors; cannabis use was assessed every four months post-baseline for 12 months. Primary analyses involved multivariable modified Poisson regressions and survival analysis.

**Results:**

In multivariable models, youth who reported lifetime cannabis use (n = 188, 48.1 %) were older, reported alcohol use and positive cannabis use expectancies. Greater self-control and self-concept were associated with lower likelihood of lifetime cannabis use. Youth who initiated cannabis during the 12-month follow-up (n = 30, 14.8 %) tended to be older, White/non-Latinx, and to report more psychiatric symptoms (posttraumatic stress, externalizing, internalizing, and affect dysregulation), delinquent behavior, lower levels of self-control, poorer self-concept, greater drug use intentions and positive cannabis expectancies. In the multivariable survival analysis, affect dysregulation, internalizing symptoms, and more positive cannabis expectancies remained independently and positively associated with cannabis initiation.

**Conclusions:**

There is a critical and unique window of opportunity to prevent cannabis use initiation among first-time justice-involved youth. Research is needed to determine whether brief interventions that aim to modify expectancies about cannabis use reduce rates of cannabis initiation in this underserved population.

## 1. Introduction

Cannabis use is on the rise, among some groups of US adolescents, due to increased availability, less overall negative perceptions, and a proliferation of e-cigarettes and vaping (Miech et al., 2019). Recent population studies show rates of use in 8^th^ and 10^th^ grades at 15 % and 34 % respectively (Miech et al., 2019). Past-year cannabis use among justice-involved youth (JIY) steadily increased between 2002–2017 (Vaughn et al., 2020) and JIY report higher rates of cannabis use (community-supervised and detained at 48 % and 54 %, respectively) than their same-age non-justice-involved peers; often starting cannabis use by age 13 (Grigorenko et al., 2015; Tolou-Shams et al., 2020).

As part of the fourth wave of juvenile justice reform (Weiss, 2013), legislation has increasingly moved toward diverting youth from detention to community supervision. System advances (Grisso, 2007; Grisso et al., 2001) including implementation of specific behavioral health screening tools for youth in detention and on probation increased identification of youth with treatment needs. Research to identify feasible and acceptable substance use interventions to implement and sustain *within* juvenile justice settings to prevent or decrease substance use is emerging (Knight et al., 2016), but in tremendous need given the shortage of such services (Funk et al., 2020). Efficacious substance use interventions for JIY include family, are intensive, and typically address secondary or tertiary prevention of substance use (e.g., Multisystemic Therapy; Henggeler et al., 1992); these are not typically feasible for implementation within busy, often overburdened and under-resourced juvenile justice settings, yet research on brief substance use prevention interventions for JIY is lacking. Individual level, modifiable factors that can be incorporated into brief interventions and feasibly delivered within juvenile justice settings to prevent and/or reduce youth substance use must be identified (Dauria et al., 2018).

Brief, empirically-supported substance use (alcohol and cannabis) interventions with adolescents/young adults (e.g., college students) focus on addressing social attitudes, beliefs, and cognitions (e.g., expectancies of substance use) and enhancing motivation to abstain from or reduce use (Borsari et al., 2007; Lee et al., 2013). Research with JIY highlights increased likelihood of substance use secondary to psychiatric symptoms, trauma exposure and symptoms, chronic absenteeism/truancy and family factors (e.g., parental monitoring, communication; Tolou-Shams et al., 2019). But, data on social cognitive influences on substance use among JIY are limited. For example, data on cannabis use expectancies with JIY are limited to a single, small detained sample in one U.S. state (Torrealday et al., 2008). Findings suggest negative cannabis use expectancies are associated with less cannabis use, while positive expectancies are unrelated. The authors posit consequences associated with use may be more salient for youth completing these measures while detained, and different associations regarding positive expectancies may have emerged if measured outside detention. Of note, the negative expectancies subscale had very low internal consistency, thus replication of their findings with other JIY samples is warranted. Other adolescent studies show negative expectancies associated with cannabis use among Black females (who are disproportionately represented in the juvenile justice system) are related to less cannabis use over time (Walther et al., 2019) and among a racially and ethnically diverse U.S. high school student sample changes in positive substance use expectancies most saliently predicted substance use onset and changes in negative expectancies was associated with onset of cannabis use only (Montes et al., 2019). Brief individual interventions addressing substance use motivations and expectancies have been successful in reducing adolescent cannabis use (Martin and Copeland, 2008; Walker et al., 2011); however, research on preventing initiation through brief intervention and among JIY is nascent. Extension of expectancies research with JIY samples is necessary, particularly using prospective data and examining the role of positive expectancies and cannabis use outside detention when there is greater opportunity for use.

Studies of school-based and general adolescent samples have also demonstrated the importance of understanding reasons for and protective factors against cannabis use. Data from the Monitoring the Future Survey examining past 10-year trends demonstrates adolescents cite more coping-related reasons than any other motivations for use (Patrick et al., 2019). Individual factors that positively influence social cognition and behaviors (e.g., self-control, self-concept) appear to buffer against substance use among early adolescents in public school (Wills and Ainette, 2010), and higher self-esteem is associated with less substance use (and delinquency) among Black adolescents exposed to community violence and with high family stress (Voisin et al., 2020). Enhanced emotion regulation skills, which are influenced by social cognitive factors (Tamir and Krauss, 2011), are also protective against cannabis use initiation among Black adolescents (Kliewer and Parham, 2019). Justice-involved youth, who experience high rates of trauma, poverty, stigma and discrimination, may cite multiple reasons to use cannabis as a coping strategy, however, research in this area is lacking.

### 1.1. Current study

Understanding how individual level, substance-related attitudes, beliefs and social cognitions influence JIY’s cannabis use, while accounting for known factors associated with increased likelihood of use, such as psychiatric symptoms (including trauma and affect dysregulation), other substance use (e.g., alcohol), and externalizing behaviors, is key to shaping the development of feasible systems-embedded brief substance use prevention interventions. Identifying individual social cognitive factors that might protect against cannabis use initiation in first-time JIY allows incorporation of a strengths versus deficit framework; a theoretical approach still largely lacking in the study of cannabis use and juvenile justice.

In this prospective cohort study of first-time JIY, we aimed to understand rates of early onset cannabis use (<13 years of age) and individual level factors associated with early onset use and new initiation in the 12 months after first court contact. We hypothesized more psychiatric symptoms, other substance use, pro-cannabis use beliefs, attitudes and intentions, and lower self-concept and less self-control would be associated with early onset use and new initiation over follow-up.

## 2. Method

### 2.1. Participants and procedure

**Fig. 1.**
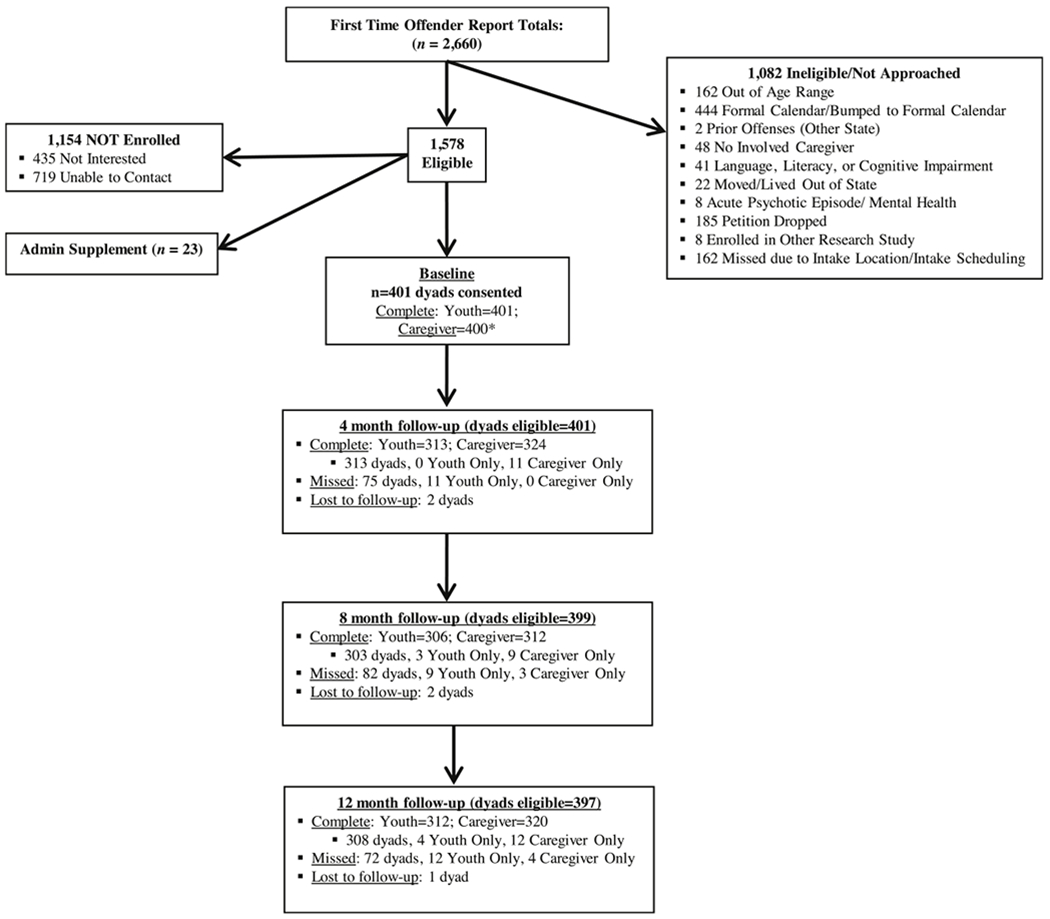
Participant retention flowchart. Note. dyad = youth and caregiver completed or missed assessment; youth only = youth completed or missed assessment but caregiver did not; caregiver only = caregiver completed or missed assessment but youth did not. *Baseline assessment was not completed for 1 caregiver, so this dyad is not part of the longitudinal sample for the current study

Eligible youth: 1) had been in contact with the court for the first time within the past 30 days; 2) had an open status (i.e., offense due to being under <18 years, such as truancy) and/or delinquent petition (i.e., illicit act regardless of age, such as assault) filed through a large family court in the northeastern U.S.; and 3) were living in the community. Exclusion criteria included being younger than 12 or older than 18, having a prior offense at time of recruitment, cognitive impairment that would impede ability to complete assessments, caregiver’s unwillingness to participate, and/or if the caregiver and youth had not lived in the same household for at least the prior six months. Court staff estimates and records indicated approximately 50 % of the 4800 juveniles seen at the court during the enrollment period (2014–2016) were potentially eligible. Eligible youth and caregivers were approached by research staff at their first court appointment (after receiving a study flyer by mail) and those interested were screened for eligibility in a private setting at the courthouse. Study consent (assent for youth) and assessments were completed in off-site, private spaces (e.g., at the home, private community space, or research lab). Tablet-based, audio-assisted computerized assessments in English (and Spanish for caregivers) took less than 2 h to complete. Follow-up assessments were conducted every four months post-baseline for two years. The current study uses data from the baseline, 4-, 8-month, and 12-month follow-up assessments (see Fig. 1 for retention). The Principal Investigator’s university and collaborating sites’ Institutional Review Boards approved all study procedures. Additional methods are described elsewhere (Tolou-Shams et al., 2020).

### 2.2. Measures

#### 2.2.1. Independent variables (baseline)

**Demographics** were reported by youth and caregivers, including age, gender, race, and ethnicity.

##### Psychiatric.

*Trauma exposure and posttraumatic stress symptoms were assessed using the* 9-item National Stressful Events Survey PTSD Short Scale (NSESSS; Kilpatrick et al., 2013). Averaged, higher total scores indicate greater symptom severity (1=*not at all* to 5=*extremely*). We added a response option (6=“I have never experienced a stressful event”) to identify youth with no trauma exposure; if youth endorsed this for any item, the entire scale was recoded as missing. Prorated scores were calculated when no more than two items were left unanswered (sum of items answered x total number of items on measure/number of items answered, rounded to the nearest whole number).

*Delinquency* was measured using the National Youth Survey Self-Reported Delinquency (Thornberry and Krohn, 2000) scale, a well-validated 40-item self-report measure of delinquent acts (e.g., larceny, fighting, selling drugs). General Delinquency subscale scores (0–23) were used; higher scores indicate greater number of delinquent acts (in the past 120 days) (α = 0.98).^1^

###### Affect dysregulation.

Youth responded to six items adapted from the Structured Interview for Disorders for Extreme Stress (Brown et al., 2012) on a scale of 1 (*not at all*) to 4 (*often*) with higher total summed scores reflecting greater affect dysregulation (α = .79).

###### Internalizing and externalizing symptoms.

Using the 148-item Behavior Assessment System for Children-2, caregivers completed the Parent Rating Scales-Adolescent regarding externalizing symptoms (α = 0.78) and youth completed the Self-Report of Personality (Reynolds and Kamphaus, 2006) regarding internalizing symptoms (α = 0.98). Summed raw scores are converted to standardized T-scores and categorized as: 59 and below (“within normal limits” or WNL; no follow-up needed), 60–69 (“at-risk”; some degree of follow-up needed), and 70 and above (“clinical range”; clinical intervention needed).

###### Alcohol and other drug use.

Youth reported whether they ever used alcohol or other drugs (e.g., cocaine) during their lifetime (yes/no) on the Adolescent Risk Behavior Assessment (ARBA; Donenberg et al., 2001) that has been used extensively to measure substance use in JIY samples (e.g., Conrad et al., 2017; Hoskins et al., 2019; Tolou-Shams et al., 2017).

##### Substance use related attitudes, beliefs and cognitions.

###### Importance of not using and intention to use drugs.

Youth responded to 2 items on the ARBA (Donenberg et al., 2001) asking “how important is it for you not to use drugs” (1=*not important* to 10=*very important)* and “how likely is it that you will use drugs in the future” (1=*unlikely* to 10=*very likely)*.

*Drinking/drug use beliefs* were assessed using four items, on a 5-point scale (1=*strongly disagree* to 5=*strongly agree*) modeled from prior research on beliefs about youth smoking (Kodl and Mermelstein, 2004). Summed, higher scores reflect more pro-drinking and drug use beliefs (α = 0.76).

*Cannabis expectancies* were assessed using the 6-item Marijuana Effect Expectancy Questionnaire, Brief (Torrealday et al., 2008) that includes positive (α = .89) and negative (α = .48) expectancies subscales with item responses of 1 (*disagree strongly*) to 5 (*strongly agree)*. Subscale summed higher scores (range 5–15) indicate greater expectancies. Due to low internal consistency of the negative expectancies scale in our sample [consistent with the initial validation paper (Torrealday et al., 2008)] we only included positive expectancies.

##### Protective factors.

###### Self-control and self-concept.

The 62-item Youth Resiliency: Assessing Developmental Strengths Scale (YRADS) (Donnon and Hammond, 2007a) assesses youth internal and external resiliency factors and developmental strengths. The YRADS has been validated with large, general adolescent samples (Donnon and Hammond, 2007b). For this study, we included internal resiliency subscales of self-control (4 items regarding self-restraint and resistance skills, α = .78) and self-concept (6 items regarding self-efficacy, self-esteem and decision-making, α = .86).

#### 2.2.2. Dependent variables

Three variables were derived from youth baseline self-report (lifetime and early onset use) and 3 follow-up assessments (every 4 months) over a 12-month period (new initiation). At baseline, youth reported via the ARBA (Donenberg et al., 2001) whether they ever smoked “any form of marijuana (e.g. pot, weed, blunts, hashish, grass or ganja)” ??in their lifetime and how old they were the first time they did. At follow-up assessments, youth reported how frequently they used cannabis over the past 120 days (i.e., since prior assessment). **Lifetime Cannabis use.** Youth who reported ever having used cannabis at baseline were coded as yes for lifetime use. **Early Onset of Cannabis Use.** Youth who used cannabis prior to age 13 at baseline were considered to have early onset use. **New Initiation of Cannabis Use.** Youth who had no lifetime use at baseline but reported cannabis use during the 12-month follow-up period were coded as new initiation.

#### 2.2.3. Plan of analysis

Descriptive statistics were examined at baseline. Next, we determined factors associated with lifetime cannabis use reported at baseline using bivariable measures of association (i.e., chi-square tests/fisher exact tests for categorical independent variables; *t*-tests for continuous variables). Third, among youth who reported lifetime cannabis use at baseline, we compared those who did and did not report early onset use at baseline. Fourth, we conducted modified Poisson regression to determine the independent associations between baseline factors and two primary outcomes: (1) lifetime cannabis use (yes/no) reported in the entire sample, and (2) early onset use (yes/no) in the subset of participants who reported baseline lifetime cannabis use. Modified Poisson regression is appropriate for non-rare dichotomous outcomes (Zou, 2004). Covariates were selected for inclusion in the multivariable models based on the standard cut-off rule of *p* < 0.05 in bivariable analyses except age, gender, and race/ethnicity, which were included in all models. We created final multivariable models using a sequential backwards selection approach, in which variables with the largest *p*-values were removed sequentially, with the final model having the lowest AIC.

Next, among youth who did not report lifetime cannabis use at baseline (all of whom had at least one follow-up observation), we compared baseline factors associated with cannabis use initiation over follow-up using the same methods as described above. We then conducted a survival analysis using Cox proportional hazards regression to determine baseline factors associated with time to cannabis use initiation among youth who reported no lifetime cannabis use at baseline. We estimated the length of follow-up by calculating the difference between the interview date during which the first instance of cannabis use was reported and the interview date of the baseline survey. All covariates were time-updated as appropriate. We used the Breslow method to handle ties in the timing of reported outcome events. All variables significant at *p* < 0.05 in bivariable survival analyses were included in the multivariable Cox proportional hazards regression model; we also included age, gender, and race/ethnicity and obtained a final model using a sequential backwards selection procedure, as above. We tested whether the proportional hazards assumption was met in all analyses using the ASSESS statement in PROC PHREG with the option PH (Lin et al., 1993). We conducted multiple imputation by fully conditional specification with the number of imputations set to 20 to account for covariates with missing data, based on the assumption those missing covariates were missing at random (White and Royston, 2009). We then conducted multivariable survival analysis based on the fully imputed datasets.

## 3. Results

### 3.1. Participants

Participants were 391 first-time JIY and an involved caregiver (Table 1). Youth were on average 14.5 years old (*SD* = 1.5 years), predominantly male (57.3 %), and racially and ethnically diverse (32.0 % White, 11.0 % Black, 14.8 % Other/Multi-racial, 41.9 % Latinx).

### 3.2. Baseline factors related to lifetime Cannabis use

Of the 391 participants, 188 (48 %) reported lifetime cannabis use at baseline and were more likely to be older, non-Latinx, and charged with a delinquent offense, than youth who had never used cannabis (Table 2). Youth with a lifetime history of cannabis use reported significantly more posttraumatic stress symptoms, delinquent behavior, affect dysregulation, and externalizing symptoms than youth who had never used cannabis. They were also more likely to endorse lifetime alcohol and other drug use, to rate the importance of not using drugs lower, to have greater drug use intentions and pro-drinking/drug use beliefs, and to have more positive expectancies for cannabis use. Youth who had never used cannabis prior to baseline also had significantly higher levels of self-control and self-concept.

### 3.3. Baseline factors related to early onset of Cannabis use

Youth who reported using cannabis for the first time before 13 years of age (*n* = 105, 56 %) were significantly younger, engaged in more delinquent behavior, and reported greater drug use intentions and more pro-drinking/drug use beliefs (Table 2).

### 3.4. Multivariable analyses of lifetime and early onset of Cannabis use

Compared to participants who reported no lifetime cannabis use at baseline, those who did were more likely to be older, report lifetime alcohol use, and endorse more positive cannabis expectancies (Table 3). Higher levels of self-control and self-concept also remained associated with a lower likelihood of lifetime cannabis use. In the final regression model, only younger age was independently associated with early onset use.

### 3.5. Baseline factors related to Cannabis use initiation

Among youth who had never used cannabis prior to baseline (*n* = 203, 52 %), we examined factors related to cannabis use initiation during the 12 months following first court contact (Table 2). Youth who initiated cannabis use (*n* = 30, 15 %) were significantly older at baseline and more likely to be White, non-Latinx, had higher levels of baseline posttraumatic stress symptoms, delinquent behavior, affect dysregulation, and externalizing symptoms, and fewer internalizing symptoms. Youth who newly initiated cannabis use reported greater drug use intentions and more positive cannabis expectancies, as well as less self-control and self-concept at baseline.

### 3.6. Survival analyses

Over the 12-month follow-up, the incidence rate of cannabis use initiation was 19.5 per 100 person-years (95 % CI: 13.4–27.6). All covariates met the proportional hazards assumption except race/ethnicity, which might be due to small cell sizes across levels of this covariate. In bivariable survival analyses, higher levels of posttraumatic stress symptoms, delinquent behavior, affect dysregulation, internalizing symptoms, and externalizing symptoms were all associated with an increased hazard of cannabis initiation over follow-up (all *p* < .05, see Table 4). Consistent with the 12-month initiation analysis, youth who reported greater drug use intentions, more positive cannabis expectancies, and less self-control and self-concept had a greater hazard of cannabis initiation over follow-up. In the final multivariable Cox proportional hazards model (Table 4), affect dysregulation (*p* = .003), internalizing symptoms (*p* = .019), and positive cannabis expectancies (*p* = .001) remained positively associated with cannabis initiation; externalizing symptoms was marginally significant (*p* = .078).

## 4. Discussion

Reducing early initiation of cannabis use is key to preventing negative long-term health and associated psychosocial consequences (National Institute on Drug Abuse, 2020; Hawes et al., 2019; Savage et al., 2017). In this large sample of first-time JIY, rates of early onset cannabis use were high and 15 % of youth newly initiated cannabis use in the year following first justice contact. Youth’s internal distress, affect dysregulation, and positive expectancies about cannabis use drove new initiation, even after accounting for known associated factors (e.g., other substance use, trauma, delinquent acts). The justice system largely focuses on interventions to address co-occurring mental health and delinquent behavior, primarily through group or family-based intervention, but our data suggest there is a critical and unique window of opportunity to prevent cannabis use initiation among youth by addressing internalizing symptoms, teaching emotion regulation skills, and modifying expectancies. Such interventions can be brief and feasible to implement within existing individual-based court and justice-related services (e.g., conducted as part of court or probation routine individual intake and screenings/assessments). Since adolescent cannabis use can be associated with future worse public health and legal outcomes, developing effective brief primary prevention interventions for JIY is critical; these are not mutually exclusive from essential development and empirical testing of structural-level public health and legal policy interventions to delay or reduce JIY substance use.

Only two studies have tested brief interventions to reduce substance use among justice-involved or diverted truant populations (Dembo et al., 2016a, 2016b; Spirito et al., 2018). Spirito and colleagues (2018) tested the preliminary efficacy of a combined family-based (Family Check-Up; FCU) and individual adolescent based brief motivational enhancement therapy (MET) intervention (one 90-minute session and one 30-minute booster); the latter targeting adolescent substance use related attitudes, beliefs and norms and demonstrating feasibility, acceptability and reductions in youth cannabis use at 3 month follow-up (Spirito et al., 2018). Dembo and colleagues (Dembo et al., 2014) tested the efficacy of a brief intervention (BI) with youth and parents (three 75-minute sessions; two with youth, one with parent) compared to youth-only BI and Standard Truancy Services in reducing cannabis use and sexual risk behavior over 12 months. No significant intervention effects were found; however, the authors note certain subgroups showed differential response to the intervention (e.g., those with attention deficit hyperactivity disorder symptoms). Although mixed in success, both studies addressed individual level factors commonly associated with increased likelihood of substance use among JIY (e.g., co-occurring psychiatric needs, impulsivity, delinquent behaviors, trauma symptoms).

Our data suggest with first-time JIY who have not initiated use, a brief individual youth intervention targeting internalizing symptoms, emotion regulation skills, and cannabis use expectancies is important for future intervention development and testing. Single session interventions (SSIs) are a cost-effective and feasible way to address youth internalizing symptoms (anxiety and depression) and increase access to mental health interventions for underserved (e.g., rural) youth (Schleider and Weisz, 2018; Schleider et al., 2019b, 2019a). SSIs focused on motivational enhancement therapy for sexual risk reduction (incorporating substance use content) have been feasible and acceptable to deliver to large numbers of detained youth (Schmiege et al., 2021). The concept of SSIs has yet to be explored for substance use prevention among JIY, but our study suggests a SSI addressing internalizing symptoms, emotion regulation, and cannabis use expectancies and intentions may be efficacious in delaying or preventing cannabis use initiation, both of which have significant positive public health implications (National Institute on Drug Abuse, 2020). SSIs could also be developed to shift expectancies and intentions about continued use for those with early onset, who are at greater risk for worse outcomes due to being younger upon first using and greater likelihood of continued use and consequences. Our results suggest incorporating alcohol use content might also be important for those already using cannabis at first-time justice contact. SSIs are also likely more feasible to implement within real-world settings already serving JIY (e.g., courts, probation) and have strong potential to address a highly concerning gap in access to substance use intervention for community-supervised JIY (Funk et al., 2020).

One possible approach for substance use SSIs is motivational interviewing (MI), a communication technique used to reduce alcohol and cannabis use among school-mandated college students (Borsari et al., 2016) and in two studies of general substance using adolescent populations (Martin and Copeland, 2008; Walker et al., 2011); however, the limited data available suggest MI for universal prevention may not be as effective (McCambridge et al., 2011). Adult criminal justice systems are incorporating MI techniques through digital health interventions to reduce substance use and in staff trainings to promote overall harm reduction and associated consequences, but studies are with those already using substances. Our data suggests focusing on youth’s internal distress (including emotion dysregulation), and cannabis use expectancies, (particularly positive expectancies), *for those in first-time legal contact and not yet using*, could be an important focus for prevention efforts. Depending on resources and time, interventions could be delivered in-person or through digital health technology (Dauria et al., 2018; Schleider and Weisz, 2018; Schleider et al., 2019a).

### 4.1. Strengths and limitations

The current study had several noteworthy strengths and limitations. Youth were sampled from a single-family court in the northeast U.S., and there was a high proportion of Latinx youth and families; future research is needed to determine generalizability of the findings to youth in other regions of the U.S. and internationally, and to non-Latinx youth and families. However, given that most studies of JIY include predominantly male samples, a key strength was that almost half of our study sample was female. The longitudinal design and use of empirically validated assessments are also study strengths. Youth self-report of substance use is potentially a limitation, particularly if youth were motivated to underreport use due to their justice involvement; however, this seems unlikely given high rates of self-reported cannabis use.

### 4.2. Conclusions

First juvenile court contact represents a critical point-in-time to deliver and test brief substance use prevention interventions addressing co-occurring internalizing symptoms, particularly given the dearth of available and accessible substance use interventions for JIY. Such interventions could potentially delay cannabis use initiation and/or reduce use, thereby contributing to improved public health and legal outcomes. Development and testing of such brief intervention modalities and studying factors associated with their successful implementation in real-world justice settings is an important next step for the field.

## Figures and Tables

**Table 1:** Demographic characteristics of justice-involved youth and caregiver dyads recruited from a family court in the northeastern region of the United States.

Variable	Youth (*n* = 391)	Caregivers (*n* = 391)
Mean age at baseline, in years (SD)	14.5 (1.5)	41.0 (7.2)
Female, *n* (%)	167 (42.7)	341 (87.2)
Race/Ethnicity, *n* (%)		
White, non-Latinx	125 (32.0)	168 (43.0)
Black, non-Latinx	43 (11.0)	34 (8.7)
Multi-racial/Other, non-Latinx	58 (14.8)	54 (13.8)
Latinx	164 (41.9)	132 (33.8)
Charge type, *n* (%)		
Status offense	191 (48.0)	–
Delinquent offense	200 (51.2)	–
Employment, *n* (%)		
Full-time	7 (1.8)	140 (35.8)
Part-time	30 (7.7)	62 (15.9)
Unemployed	341 (87.2)	189 (48.3)
Relationship to youth, *n* (%)		
Birth parent	–	362 (92.6)
Step-parent	–	6 (1.5)
Adoptive parent	–	11 (2.8)
Foster parent	–	1 (0.3)
Aunt/Uncle	–	3 (1.8)
Grandparent	–	7 (1.8)
Other	–	1 (0.3)
Marital Status, *n* (%)		
Single, never married	–	148 (37.9)
Married	–	103 (26.3)
Separated/divorced	–	99 (25.3)
Living with partner	–	25 (6.4)
Other	–	15 (3.8)

**Table 2:** Individual level factors related to lifetime, early onset, and new initiation of cannabis use among justice-involved youth.

		Lifetime Use			Early Onset Use			12-Month New Initiation		
	Overall (*N* = 391)	Yes (*N* = 188)	No (*N* = 203)	*p*-value	Yes (*N* = 105)	No (*N* = 81)	*p*-value	Yes (*N* = 30)	No (*N* = 173)	*p*-value
**Demographic**										
Age, *M* (*SD*)	14.5 (1.5)	15.2 (1.4)	13.9 (1.4)	<.0001	14.6 (1.3)	16.0 (1.0)	<.0001	14.4 (1.3)	13.8 (1.5)	.039
Gender, *N* (%)				.119			.507			.731
Male	220 (56.3)	99 (52.7)	121 (59.6)		57 (54.3)	40 (49.4)		19 (63.3)	102 (59.0)	
Female	168 (43.0)	89 (47.3)	79 (38.9)		48 (45.7)	41 (50.6)		11 (36.7)	68 (39.3)	
Race/Ethnicity, *N* (%)				.184			.484			.033
White, non-Latinx	125 (32.0)	68 (36.2)	57 (28.1)		41 (39.1)	27 (33.3)		12 (40.0)	45 (26.0)	
Black, non-Latinx	43 (11.0)	19 (10.1)	24 (11.8)		9 (8.6)	9 (11.1)		2 (6.7)	22 (12.7)	
Other/Multi-racial, non-Latinx	58 (14.8)	31 (16.5)	27 (13.3)		20 (19.1)	11 (13.6)		0 (0.0)	27 (15.6)	
Latinx	164 (41.9)	70 (37.2)	94 (46.3)		35 (33.3)	34 (42.0)		16 (53.3)	78 (45.1)	
Offense, *N* (%)				.046			.202			.966
Status	191 (48.9)	82 (43.6)	109 (53.7)		50 (47.6)	31 (38.3)		16 (53.3)	93 (53.8)	
Delinquent	200 (51.2)	106 (56.4)	94 (46.3)		55 (52.4)	50 (61.7)		14 (46.7)	80 (46.2)	
**Mental Health**										
Trauma Exposure, *N* (%)				.131			.843			.980
Yes	312 (79.8)	156 (83.0)	156 (76.9)		17 (16.2)	14 (17.3)		23 (76.7)	133 (76.9)	
No	79 (20.2)	32 (17.0)	47 (23.2)		88 (83.8)	67 (82.7)		7 (23.3)	40 (23.1)	
Posttraumatic Stress Symptoms, *M* (*SD*)	1.1 (1.1)	1.3 (1.1)	1.0 (1.0)	.022	1.4 (1.1)	1.2 (1.0)	.160	1.4 (1.0)	0.9 (1.0)	.029
Delinquency, *M* (*SD*)	2.1 (2.7)	3.3 (3.2)	1.1 (1.6)	<.0001	3.8 (3.4)	2.6 (3.0)	.014	1.8 (1.9)	0.9 (1.4)	.028
Affect Dysregulation, *M* (*SD*)	12.7 (4.3)	13.4 (4.3)	12.1 (4.3)	.002	13.5 (4.3)	13.4 (4.3)	.860	14.1 (4.7)	11.7 (4.1)	.006
Internalizing Symptoms, *N* (%)				.194			.552			.015
Normal	275 (72.9)	126 (68.9)	149 (76.8)		70 (66.7)	55 (71.4)		17 (56.7)	132 (76.3)	
At-Risk	46 (12.2)	27 (14.8)	19 (9.8)		15 (14.3)	12 (15.6)		7 (23.3)	12 (6.9)	
Clinical	56 (14.9)	30 (16.4)	26 (13.4)		20 (19.1)	10 (13.0)		4 (13.3)	22 (12.7)	
Externalizing Symptoms, *N* (%)				.016			.175			.040
Normal	216 (55.7)	90 (48.1)	126 (62.7)		46 (44.2)	44 (54.3)		14 (46.7)	112 (65.5)	
At-Risk	81 (20.9)	46 (24.6)	35 (17.4)		25 (24.0)	21 (25.9)		5 (16.7)	30 (17.3)	
Clinical	90 (23.5)	51 (27.3)	40 (19.9)		33 (31.7)	16 (19.8)		11 (36.7)	29 (16.8)	
**Substance Use**										
Baseline Alcohol Use, *N* (%)				<.0001			.291			.712
Yes	129 (33.0)	113 (60.1)	16 (7.9)		67 (64.4)	46 (56.8)		3 (10.0)	13 (7.5)	
No	260 (66.5)	74 (39.4)	186 (91.6)		37 (35.6)	35 (43.2)		27 (90.0)	159 (91.9)	
Lifetime Drug Use, *N* (%)				<.0001			.605			1.000
Yes	52 (13.5)	49 (26.6)	3 (1.5)		29 (28.4)	20 (25.0)		0 (0.0)	3 (1.7)	
No	332 (86.5)	135 (73.4)	197 (98.5)		73 (71.6)	60 (75.0)		29 (96.7)	168 (97.1)	
Importance of Not Using Drugs, *M* (*SD*)	7.1 (3.4)	6.0 (3.2)	8.0 (3.4)	<.0001	5.8 (3.2)	6.4 (3.1)	.256	7.6 (3.5)	8.1 (3.4)	.535
Intention to Use Drugs	3.1 (3.1)	4.8 (3.4)	1.5 (1.5)	<.0001	5.4 (3.4)	4.1 (3.1)	.009	2.0 (1.8)	1.4 (1.5)	.044
Drinking/Drug Use Beliefs	8.8 (4.3)	10.3 (4.2)	7.3 (3.8)	<.0001	11.0 (4.2)	9.4 (4.0)	.011	8.1 (3.8)	7.1 (3.8)	.209
Positive Cannabis Expectancies	10.6 (3.7)	12.2 (2.8)	8.9 (3.8)	<.0001	12.2 (2.7)	12.2 (2.9)	.924	11.0 (3.7)	8.5 (3.7)	.001
**Protective Factors**										
Self-Control	74.1 (21.0)	66.5 (19.7)	81.4 (19.7)	<.0001	66.5 (18.3)	66.5 (21.6)	.982	73.7 (23.5)	82.7 (18.7)	.024
Self-Concept	70.9 (20.1)	68.5 (20.3)	73.3 (19.7)	.020	68.6 (19.7)	67.9 (21.0)	.793	64.6 (20.3)	74.7 (19.3)	.011

Note. Total *n* for early onset use is 186 due to missing data on age of first use for 2 participants who endorsed lifetime cannabis use at baseline. Results reflect findings from bivariable measures of association (i.e., chi-square tests/fisher exact tests for categorical independent variables; *t*-tests for continuous variables).

**Table 3:** Multivariable modified Poisson regression models of factors associated with lifetime and early onset cannabis use among justice-involved youth.

	Lifetime Use				Early Onset Use			
	Beta (S.E.)	Adjusted Relative Risk (RR)	95 % CI	*p*-value	Beta (S.E.)	Adjusted Relative Risk (RR)	95 % CI	*p*-value
**Demographic**								
Age^a^	0.18 (0.07)	1.20	1.05, 1.37	0.006	−0.32 (0.08)	0.73	0.62, 0.85	0.0001
Gender								
Male	Ref				Ref			
Female	0.19 (0.17)	1.21	0.86, 1.71	0.268	−0.16 (0.21)	0.86	0.56, 1.30	0.464
Race/Ethnicity								
White, non-Latinx	Ref				Ref			
Black, non-Latinx	−0.00 (0.34)	1.00	0.51, 1.95	0.994	−0.06 (0.37)	0.94	0.45, 1.96	0.866
Other/Multi-racial, non-Latinx	−0.22 (0.21)	0.81	0.54, 1.21	0.299	−0.11 (0.24)	0.90	0.56, 1.45	0.666
Latinx	0.30 (0.26)	1.35	0.82, 2.23	0.238	0.09 (0.29)	1.09	0.62, 1.94	0.756
Offense								
Status	Ref							
Delinquent	0.06 (0.18)	1.06	0.75, 1.49	0.754	—			
**Mental Health**								
Posttraumatic Stress Symptoms ^a^	0.02 (0.09)	1.02	0.86, 1.21	0.841	—			
Externalizing Symptoms								
Normal	Ref				—			
At-Risk	0.07 (0.21)	1.08	0.71, 1.63	0.731	—			
Clinical	0.11 (0.22)	1.12	0.73, 1.72	0.601	—			
**Substance Use**								
Baseline Alcohol Use								
Yes	0.43 (0.21)	1.54	1.03, 2.31	0.036	—			
No	Ref				—			
Lifetime Drug Use								
Yes	−0.02 (0.22)	0.98	0.64, 1.50	0.922	—			
No	Ref				—			
Importance of Not Using Drugs ^a^	−0.02(0.03)	0.98	0.93, 1.04	0.577	—			
Intention to Use Drugs ^a^	0.04 (0.03)	1.04	0.97, 1.11	0.273	0.05 (0.03)	1.05	0.99, 1.13	0.114
Drinking/Drug Use Beliefs^a^	0.02 (0.02)	1.02	0.97, 1.07	0.423	0.01 (0.03)	1.01	0.96, 1.06	0.698
Positive Cannabis Expectancies^a^	0.07 (0.03)	1.07	1.00, 1.15	0.038	—			
**Protective Factors**								
Self-Control ^a^	−0.01 (0.01)	0.99	0.98, 1.00	0.025	—			
Self-Concept ^a^	0.01 (0.01)	1.01	1.00, 1.02	0.027	—			

Note. *N* = 391; Total *n* for early onset use is 186 due to missing data on age of first use for 2 participants who endorsed lifetime cannabis use at baseline. Results reflect findings from multivariable modified Poisson regression analyses. Only variables that remained in the final model are shown. — indicates variables that were not included in the final model for early onset use.

a For continuous variables, higher scores reflect higher levels of the construct, (e.g., for age, per unit older; for positive cannabis use expectancies, per unit more positive expectancies).

**Table 4:** Individual level predictors of cannabis use initiation (survival analysis results).

	Bivariable Analysis		Multivariable Analysis	
	Hazard ratio (95 % CI)	*p*-value	Hazard ratio (95 % CI)	*p*-value
Variable				
Age^a^	1.22 (0.96–1.55)	.109	1.44 (0.79–2.63)	.239
Gender (ref = Female)	0.91 (0.43–1.91)	.798	1.60 (0.47–5.48)	.454
Race (ref = White, non-Latinx)		.741		.188
Black, non-Latinx	0.44 (0.10–1.95)	.277	N/A	
Other or multiracial, non-Latinx	0.00 (0.00–0.00)	.987	N/A	
Latinx	0.81 (0.38–1.71)	.575	3.62 (1.14–11.45)	.029
Posttraumatic Stress Symptoms^a^	1.04 (1.00–1.08)	.030	0.93 (0.84–1.02)	.108
Delinquency^a^	1.26 (1.04–1.53)	.020	1.02 (0.71–1.46)	.905
Affect Dysregulation^a^	1.13 (1.04–1.22)	.003	1.38 (1.11–1.70)	.003
Internalizing Symptoms (ref = WNL)		.011		.019
At-risk	3.91 (1.61–9.49)	.003	11.18 (1.75–71.27)	.011
Clinical	1.44 (0.49–4.29)	.510	0.98 (0.16–6.10)	.985
Externalizing Symptoms (ref = WNL)		.045		.078
At-Risk	1.36 (0.49–3.79)	.557	6.66 (1.25, 35.53)	.026
Clinical	2.72 (1.23–5.99)	.013	2.31 (0.49–10.81)	.288
Intention to Use Drugs^a^	1.23 (1.04–1.45)	.013	1.30 (0.88–1.93)	.194
Positive Cannabis Expectancies^a^	1.22 (1.08–1.38)	.001	1.64 (1.22–2.21)	.001
Self-Control^a^	0.99 (0.97–1.00)	.036	1.02 (0.97–1.07)	.491
Self-Concept^a^	0.98 (0.96–1.00)	.011	1.00 (0.97–1.04)	.855

Note. N/A = not-estimable; WNL = within normal limits; - = not included in the model; Results reflect bivariable and multivariable survival analyses.

a For continuous variables, higher scores reflect higher levels of the construct, (e.g., for age, per unit older; for positive cannabis use expectancies, per unit more positive expectancies).

## Footnotes

1 The original subscale includes 24 items. Due to an error in the audio-assisted computerized assessment development, item 24, “Have you had sexual intercourse with a person who was not your serious partner when involved in a relationship?” was not administered to study participants; therefore, subscale scores range from 1–23 but still accurately indicate that greater scores represent greater number of delinquent acts.
